# Development of a strategy to control COVID-19 in hard-to-reach migrant communities: Annalisa Rosso

**DOI:** 10.1093/eurpub/ckac129.663

**Published:** 2022-10-25

**Authors:** A Rosso, A Rinaldi, D Coluzzi, F Perrelli, PA Napoli, P Villari

**Affiliations:** 1 Sapienza University of Rome, Rome, Italy; 2 Migrants’ Health Unit, Local Health Unit Roma 2, Rome, Italy

## Abstract

**Issue/Problem:**

The risk of SARS-Cov-2 infection and its adverse health effects proved to be higher among socially disadvantaged groups, including migants and ethnic minorities. Hard-to-reach (HTR) migrants, such as undocumented people, those living in informal settelments (e.g squats) or roma people have experienced severe barries to access COVID-19 information, testing and vaccination services.

**Description of the problem:**

During 2020 and 2021, the Migrants'Health Unit of Roma 2 Local Health Unit (ASL) developed different strategies to control the COVID-19 epidemics in HTR communities, addressing both the containment of clusters in informal settelments and access to COVID-19 vaccination for these population.

**Methods:**

A multicomponent and multidisciplinary strategy was implemented, based on a strong collaboration of different services across the ASL and with Non Governmental Organizations (NGOs). Starting from a mapping of the settlements and the identification of the main critical issues for the control of the epidemic in the target populations, interventions were carried out that included the involvement of NGOS in active surveillance, reporting of suspected cases of COVID-19 to the ASL and information to the communities, and the reorganization of health interventions (eg, swabs, epidemiological investigations, COVID-19 vaccinations) directly in HTR communities’ life places.

**Results:**

In the period from April 2020 to February 2021, 15 outbreaks were controlled, for a total of over 4500 persons reached, and 265 COVID-19 cases identified. From July to November 2021, vaccinations were offered in outreach or with dedicated vaccination sessions, which reached 1664 people. The intervention model, based on a deep context analysis, strong multisectoral collaboration, community involvement, lays the foundations for the design of public health strategies, not only aimed at HTR populations.

**Key messages:**

• Controlling COVID-19 in Hard- to- reach migrant populations was possible thanks to a strong collaboration between public health services and NGOs.

• Public health interventions addressed complex groups should envisage intersectoral collaborations, reorientation of services in order to meet target groups’ need and community involvement.

